# Ice-Active Substances from the Infective Juveniles of the Freeze Tolerant Entomopathogenic Nematode, *Steinernema feltiae*

**DOI:** 10.1371/journal.pone.0156502

**Published:** 2016-05-26

**Authors:** Farman Ali, David A. Wharton

**Affiliations:** Department of Zoology, University of Otago, P.O. Box 56, Dunedin, New Zealand; Michigan State University, UNITED STATES

## Abstract

*Steinernema feltiae* is a moderately freezing tolerant nematode, that can withstand intracellular ice formation. We investigated recrystallization inhibition, thermal hysteresis and ice nucleation activities in the infective juveniles of *S*. *feltiae*. Both the splat cooling assay and optical recrystallometry indicate the presence of ice active substances that inhibit recrystallization in the nematode extract. The substance is relatively heat stable and largely retains the recrystallization inhibition activity after heating. No thermal hysteresis activity was detected but the extract had a typical hexagonal crystal shape when grown from a single seed crystal and weak ice nucleation activity. An ice active substance is present in a low concentration, which may be involved in the freezing survival of this species by inhibiting ice recrystallization.

## Introduction

Cold tolerant ectotherms have evolved a number of strategies to survive low temperatures [1, 2]. Short-term freezing survival, surviving the freezing event itself, is enhanced by a slow rate of freezing protecting animals from physical damage by slowing the rate of ice crystal growth [3]. Longer-term freezing survival of organisms however, also depends upon the production of a substance that inhibits recrystallization, and/or the production of low molecular weight compounds, e.g. trehalose [4, 5]. Many cold tolerant organisms produce proteins in response to reduced temperature that help them survive freezing. Those that interact with ice could be collectively named ice active proteins [6]. Some ice active proteins have the ability to bind to the ice surface thereby affecting the formation and stability of ice. They interact in different ways with ice, assisting the organism to survive sub-zero temperatures. They either inhibit the growth of ice (antifreeze proteins: [7, 8] or trigger ice formation (ice nucleating proteins: [9]. Antifreeze proteins are more common in freeze avoiding organisms, while ice nucleating proteins are often associated with freeze-tolerant organisms [10]. Antifreeze proteins inhibit the growth of ice by producing a thermal hysteresis. Thermal hysteresis is a non-colligative property resulting in freezing point depression, in the presence of an ice crystal, without changing the melting point. The difference in freezing and melting points is the amount of thermal hysteresis [11]. Ice nucleating proteins conversely, ensure ice formation at relatively high sub-zero temperature protecting the freeze-tolerant organism from the rapid ice formation that occurs at lower freezing temperatures. Ice nucleating agents serve as nuclei for ice crystal formation and can be either external to (exogenous ice nucleation) or present within the body (endogenous ice nucleation) of the organism [12].

Some authors consider that there is a third category of ice active protein called a ‘recrystallization-inhibiting protein’, found in some freeze-tolerant animals [6]. Recrystallization is the growth of larger ice crystals at the expense of smaller ones, resulting in fewer but larger crystals. This could be damaging if it occurs in a frozen organism due to the growth of ice crystals damaging membranes or to the migration of still-liquid salty domains [13, 14]. Some animals, including nematodes, use recrystallization inhibiting proteins to inhibit recrystallization. These proteins have little or no thermal hysteresis activity but have a role in controlling the shape, formation and stability of ice crystals by inhibiting recrystallization in freeze-tolerant organisms [6]; whereas antifreeze proteins have both thermal hysteresis and recrystallization inhibition activity.

*Steinernema feltiae* is a freeze-tolerant nematode and either thermal hysteresis or recrystallization inhibition activity or both could be associated with its freezing survival. When frozen relatively rapidly *S*. *feltiae* shows modest abilities to survive freezing with a lower lethal temperature of -5°C [15]. *Steinernema carpocapsae* is the only entomopathogenic nematode so far demonstrated to have recrystallization inhibition activity [16]. No other entomopathogenic nematode has been examined for recrystallization inhibition, ice nucleation or thermal hysteresis. Therefore, this paper reports the first detailed study of recrystallization inhibition, ice nucleation and thermal hysteresis from a freeze-tolerant entomopathogenic nematode, *S*. *feltiae*.

## Materials and Methods

### Preparing nematode supernatant

*Steinernema feltiae* was reared in bee wax moth larvae, *Galleria mellonella* at 22°C. Freshly harvested third-stage infective juveniles of *S*. *feltiae* were passed through two layers of tissue paper to obtain active nematodes. Nematodes were washed in artificial tap water [17] and centrifuged to get a concentrated pellet. The weight of a 10 μl subsample was determined to calculate the total dry weight of the nematode sample. The water was then removed and the sample transferred to 1 ml buffer (25 mM Tris HCl, pH 8) in a glass homogenizer and homogenized for 15 min on ice until the nematodes disrupted completely. Protease inhibitor was not used as in previous experiments protease inhibitor itself showed some RI activity, producing misleading results. The homogenate was then centrifuged at 10,000g for 10 min and the supernatant taken. If the supernatant was still turbid it was passed through a 0.22 μm syringe filter. The supernatant was used immediately for recrystallization inhibition assays, thermal hysteresis and ice nucleation activities, or stored at −70°C for future use.

### Splat freezing assays

Recrystallization inhibition was assessed using the splat freezing technique [18] and as described by Ramløv [19]. Briefly, a 10 μl drop of sample was dropped from a height of about 2.5 m onto the polished surface of an aluminum block pre-cooled to −78°C by dry ice. A portion of the resulting thin disc of ice was transferred between two small glass coverslips to a microscope cold stage held at −20°C, mounted on a Zeiss Axiophot Photomicroscope. The temperature of the cold stage was then raised to the annealing temperature (−8°C) and the ice crystals were photographed between crossed Polaroids at the start and after 30 min of the annealing period using a Canon Powershot A640 digital camera. Ice crystal size during annealing was determined by measuring the diameters of the 10 largest crystals in the images using Axio Vision v. 4.6 software (Zeiss) run on an Insite PC. Samples demonstrating RI activity were diluted 1:1 and 1:3 with buffer and subjected to the splat freezing assay. Nematode extract was also exposed to temperatures in the range 60–80°C for one hr to test if the RI activity was due to a protein which degrades with heating.

### Optical recrystallometry

**Fig 1:**
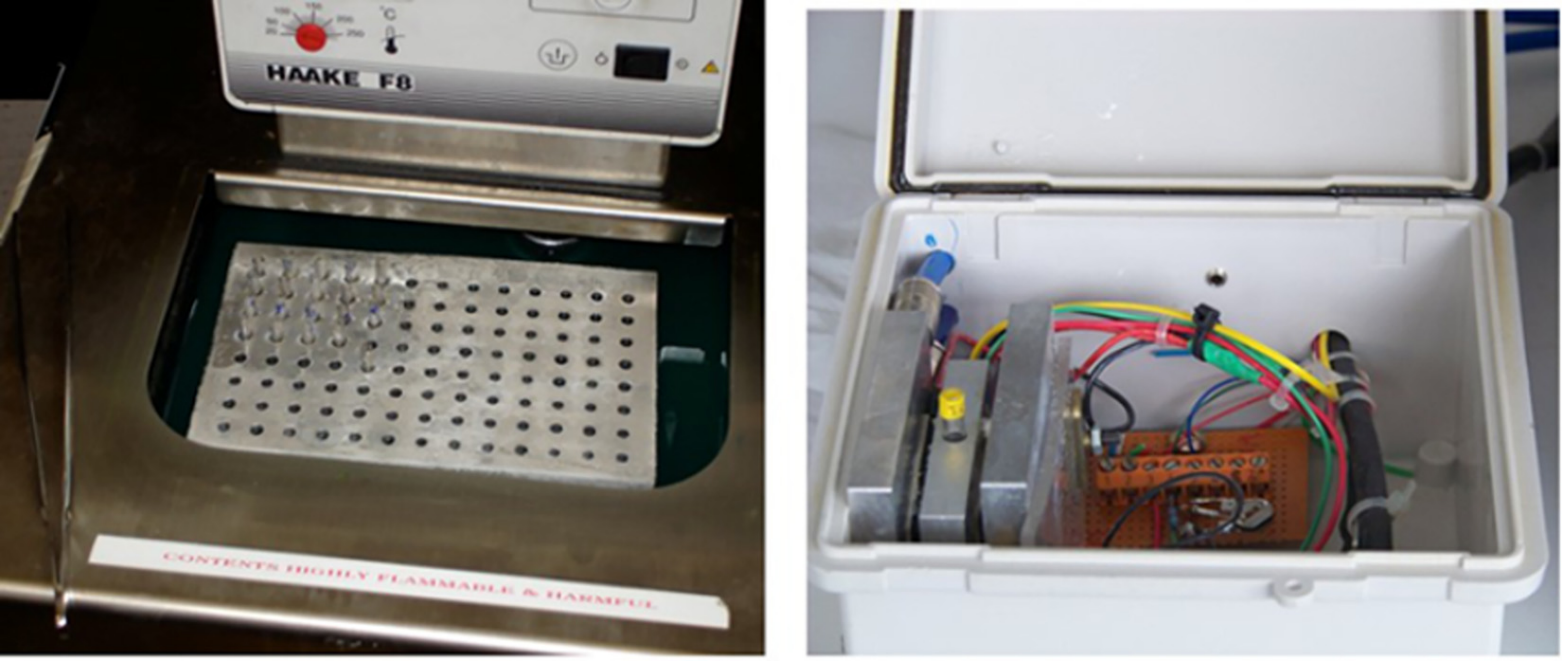
(Left) Metal rack holding the sample tubes, partly immersed in the ethanol bath of a refrigerated circulator. (Right) Sample chamber of the optical recrystallometer apparatus used for measuring RI activity.

The optical recrystallometer (Otago Osmometers: www.otago-osmometers.com) measures changes in optical transmittance of a frozen sample at a preset annealing temperature. Samples having RI activity do not change their level of optical transmittance with time whereas in samples with no RI transmittance increases with time as the ice crystals grow and scatter less light [20]. Optical recrystallometry, compared with splat freezing, is faster and a large number of samples can be processed at one time. Approximately 200 μl of each sample (nematode extracts, its dilutions and buffer) was transferred to a glass tube and frozen in an ethanol/dry ice slush at −78°C for one min. The tubes were then placed in a metal rack partly immersed in a refrigerated circulator held at −20°C (Fig 1 left) and then slowly warmed to various annealing temperatures (−6, −7, −8°C). The optical recrystallometer was calibrated with an empty tube and a tube containing a wooden skewer, to block the light path, producing readings of 100 and 0 transmittance respectively. Dry air was supplied to prevent condensation and the specimen holder of the optical recrystallometer was kept at the same annealing temperature. As soon as the annealing temperature was reached, the tubes were removed in turn, wiped with tissue and placed in the optical recrystallometer (Fig 1 right) to record the light transmittance. The readings were then taken after 1, 3 and 24 hrs of annealing at the test temperature. Each sample was replicated three times.

### Nanolitre osmometry

*Steinernema feltiae* extract was assayed for thermal hysteresis activity using a nanolitre osmometer (Otago Osmometers: www.otago-osmometers.com). Drops of each sample (nematode extract, 1000 mmolkg^-1^ standard, buffer and Milli-Q water) were transferred to mineral oil (Cargille’s A, Cedar Grove, NJ, USA) in watch glasses. The sample wells of the osmometer were filled with oil (Cargille’s B) and droplets of the samples transferred to it using a micropipette system. They were rapidly frozen by cooling the osmometer to ~ −40°C. The temperature was then raised rapidly until close to the expected melting temperature, and then slowly (0.01°C min^-1^) until the last ice crystal melted, which was taken as the melting point. Then the temperature was decreased rapidly to refreeze the sample and then increased to melt the sample to a single ice crystal. Just before the ice crystal melted, the temperature was decreased until a discernible growth of the ice crystal was observed. This was taken as the hysteresis freezing point. Thermal hysteresis was measured as the difference between melting and hysteresis freezing points. The shape of ice crystal was noted and photographed. The melting points of 1000 mmolKg^-1^ standard and Milli-Q water were used to correct observed nematode extract melting points and their osmolality calculated.

### Ice nucleation activity

Ice nucleation activity was determined using an ice nucleation spectrometer similar to that described by Wharton *et al* [21]. A 10 μl drop of nematode extract, its dilutions with buffer: 1:1, 1:3, 1:7, or buffer was placed on parafilm and drawn up into a thin-walled capillary tube. The ends of the tubes were sealed with Cargille’s A and cristaseal. Sample tubes were transferred to aluminum holders (24 samples in 4 holders), a thermocouple inserted in the middle of each holder and placed in a cooling block the temperature of which was controlled by fluid circulating from a Haake Phoenix II-C35P programmable refrigerated circulator. Thermocouples were interfaced to a Macintosh computer via a Powerlab A/D interface (Analog Digital Instruments, London). The temperature records were analyzed using a computer programme (Chart v3.2.7, Analog Digital Instruments). The temperature was lowered from 2°C to −30°C at 0.5°C min^-1^ and the temperature of crystallization (T_c_: where spontaneous freezing occurs) was read as the start of each sample exotherm.

## Results

### Recrystallization inhibition: splat freezing assays

**Fig 2:**
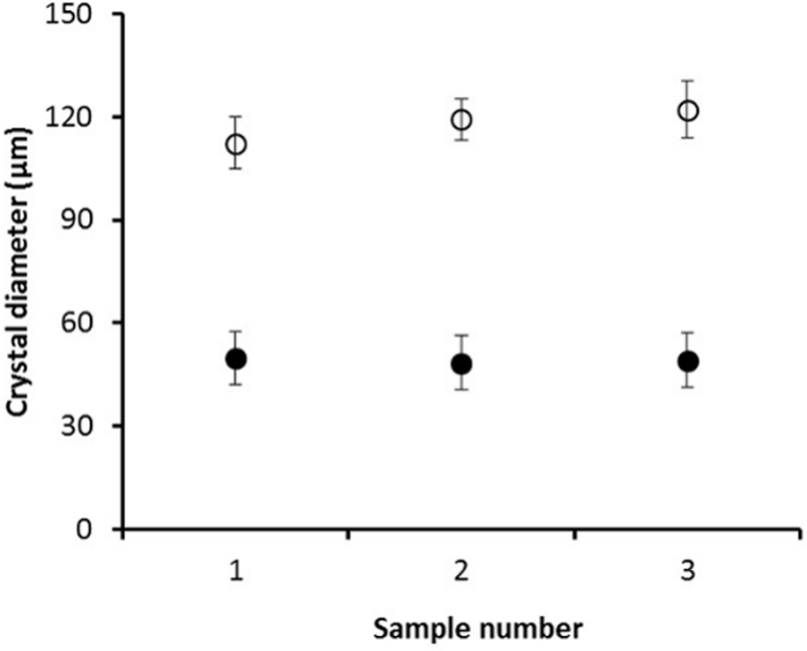
Crystal diameters of nematode extracts (closed circles) and buffer controls (open circles) after annealing at −8°C for 30 min of splat frozen samples. Means ±SD, N = 10.

**Fig 3:**
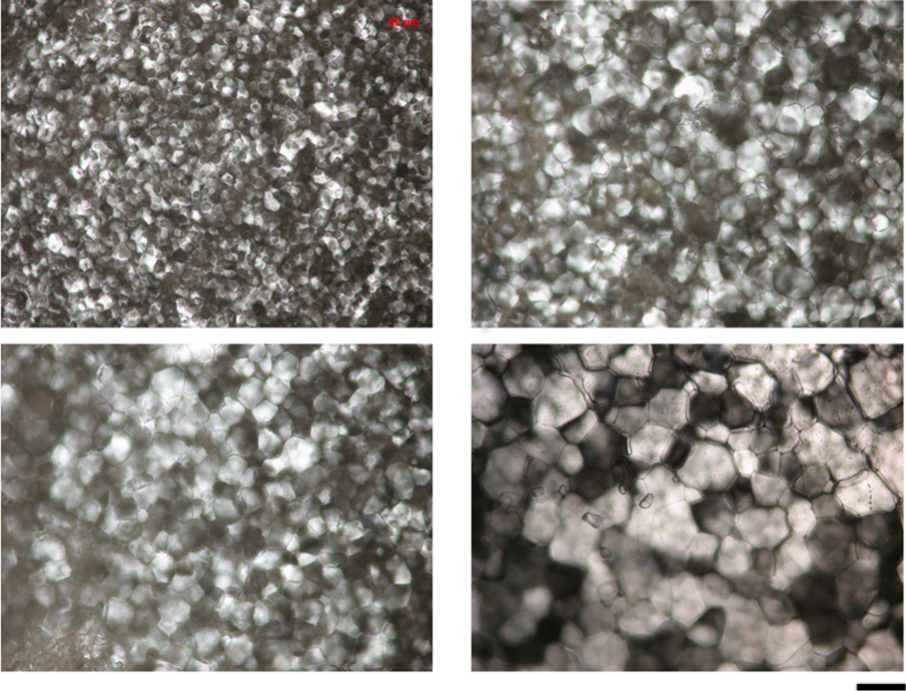
Splat frozen samples of *S*. *feltiae* extract (top row) and buffer control (25 mM Tris HCl, pH 8) (bottom row) after warming to – 8°C (Time 0 = left column) and after annealing at– 8°C for 30 min (right column). Scale bar = 100 μm.

**Fig 4:**
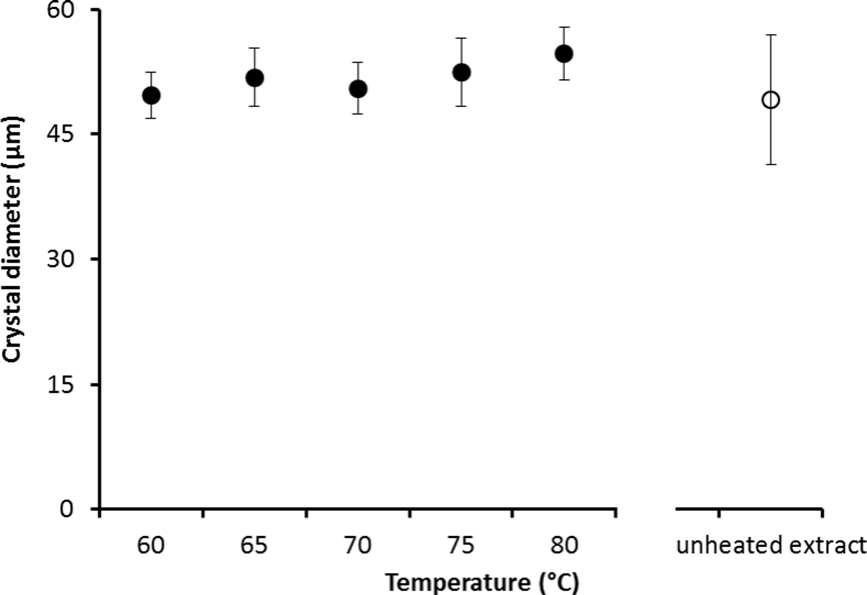
The effect of temperature on the ice crystal size of the nematode extract after heating at various temperatures for 1 hr. Means ± SD, N = 3.

*Steinernema feltiae* extract (25.3 mg dry weight/ml) showed moderate recrystallization inhibition (RI) activity. The mean crystal diameter after 30 min annealing at −8°C of nematode extract (49.1±7.84 μm, mean ± SD) was significantly smaller (~ 2.5 times, *t* = −23.4, *P* < 0.05) than that of a buffer control (117.9±7.84 μm) in the splat freezing technique (Figs 2 and 3). The effect of serial dilution on RI was significant (*F* = 271.5, *P* < 0.05), and the activity is lost (reduced to that of the buffer control) after a 1:3 dilution. However, heating did not reduce the RI activity of the nematode extract significantly (*F* = 5.4, *P* > 0.05) in terms of increase in crystal size (Fig 4). The maximum loss of RI activity is 11.4 ± 3.1%, after exposure to 80°C.

### Recrystallization inhibition: optical recrystallometry

**Fig 5:**
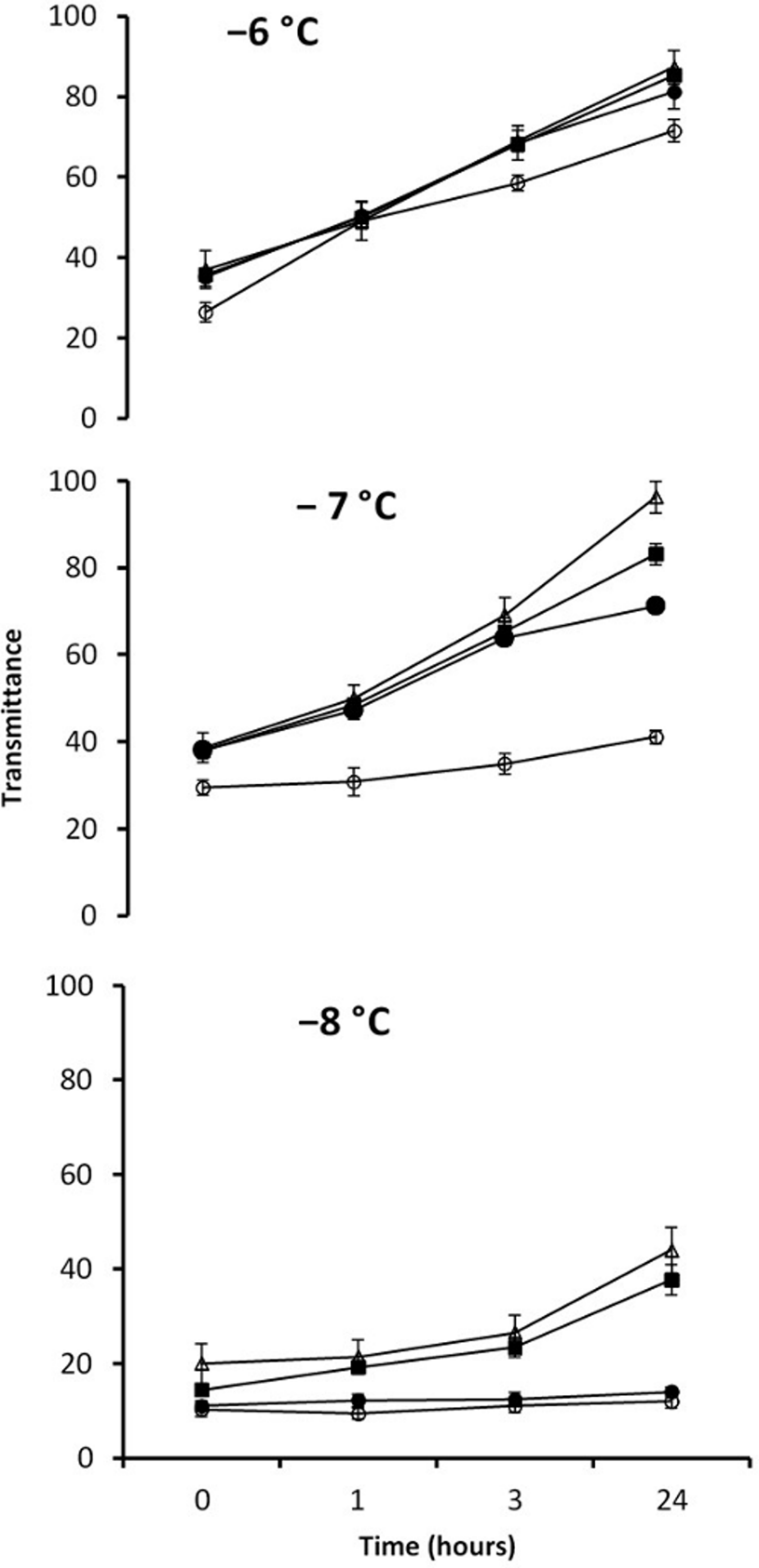
The effect of time on optical transmittance (arbitrary units) in the recrystallometer. Pure nematode extract (open circles), buffer (triangles), a 1:1 dilution of extract with buffer (closed circles) and a 1:3 dilution (squares) at annealing temperatures: -6°C (top graph), -7°C (middle graph) and -8°C (bottom graph). Means ±SD, N = 3.

In general, the pattern observed was that nematode extracts showed little or no change in optical transmittance with time, indicating RI activity; whereas in buffer, or diluted samples optical transmittance increased, indicating no RI activity. Since recrystallization is temperature sensitive, different annealing temperatures were tested. The change in the optical transmittance of the extract was less at −8°C than at −7°C and −6°C (Fig 5). Partial melting of samples occurred at −6°C and −7°C, so −8°C was chosen as the standard annealing temperature. Nematode extracts showed little change in readings between 0 and 24 hrs at −8°C compared to the buffer control. The difference between the nematode extracts and buffer samples was significant (*F* = 106.5, *P* < 0.05) but the level of transmittance in the buffer control were not significantly different (Tukey test, *P* > 0.05) from the second serial dilution (1:3) of the nematode extract.

**Fig 6:**
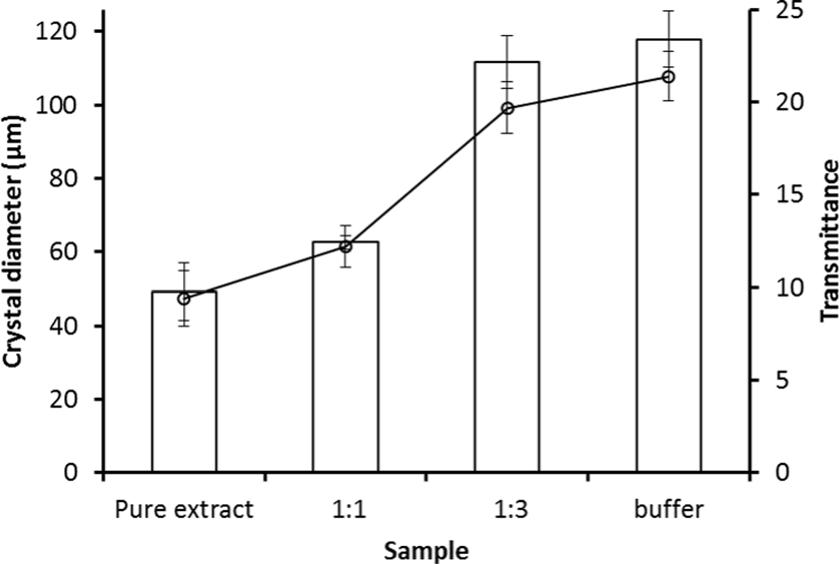
The effect of dilution of *S*. *feltiae* extracts with tris buffer on crystal diameter (bars) and optical transmittance (arbitrary units) (line) after annealing for 30 min at – 8°C. Error bars represent ±standard deviations, N = 10 for crystal diameter and 4 for optical transmittance.

A similar pattern of RI activity is shown by both the splat freezing assay and optical recrystallometry (Fig 6). This comparison has been carried out at the same annealing temperature (−8°C) but the annealing time in the splat freezing assay was 30 min and 1 hr in the optical recrystallometer. However, there was no change in the optical transmittance within the first hour of annealing (Fig 5), and so the conditions were almost the same in both sets of experiments.

### Nanolitre osmometry

**Fig 7:**
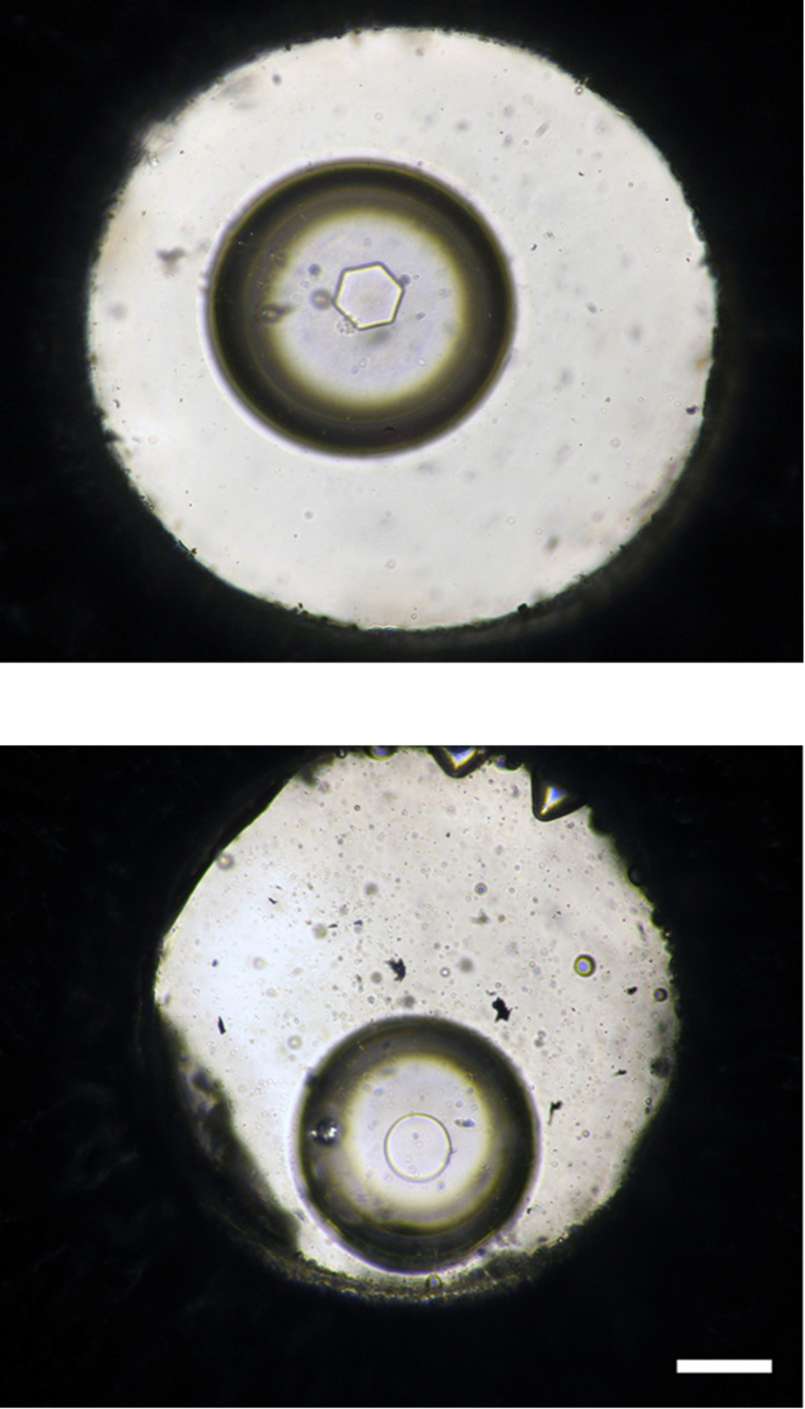
Growth of a single Ice crystal in nematode extract (top) with clear hexagonal faceting, and a disc shaped crystal in Tris HCl (bottom) as seen on a Nanolitre osmometer. Scale bar = 100 μm.

Extracts from *S*. *feltiae* showed no thermal hysteresis but had a typical hexagonal crystal shape when grown from a single seed crystal (Table 1, Fig 7). No thermal hysteresis activity or hexagonal crystal growth was detected in the 1000 mmol Kg^-1^ standard, Milli-Q water, or the buffer.

**Table 1:** Nanolitre osmometer measurements on *Steinernema feltiae* extract and controls.

Sample	Osmolality (mmol kg^-1^)	Thermal hysteresis (°C)	Ice crystal shape
***S*. *feltiae* extract**	191 ± 13	0.03	Hexagonal
**25 mM Tris HCl**	69 ± 4	0.02 ± 0.01	Disc
**MQ Water**	0	0	Disc

Means ± SE, N = 4

### Ice nucleation activity

**Fig 8:**
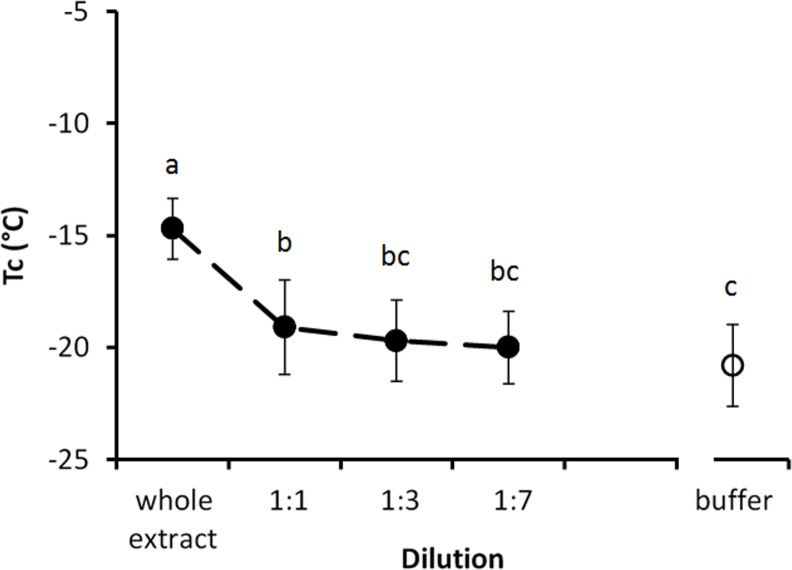
Ice nucleation activity in *S*. *feltiae* in comparison with buffer control (25 mM Tris, pH 8). Error bars are ±standard deviations, N = 24. Different small letters above the error bars represent that treatments are significantly different (*P* < 0.05).

*Steinernema feltiae* extract exhibited a weak ice nucleating activity. Spontaneous freezing (T_c_) of the extract occurred at –14.7 ± 1.37°C which was significantly different (*P* < 0.05) to that of the buffer control (–20.8 ± 1.83°C). Serial dilution had a significant effect on the activity (*F* = 43.8; *P* < 0.05) and after a 1:3 dilution it did not differ from that of the buffer control (*P* > 0.05) (Fig 8).

## Discussion

Infective juveniles of *S*. *feltiae* showed a moderate level of RI activity in the splat freezing assay. This was indicated by smaller ice crystals after the annealing period in comparison to the buffer control. Heating the extract did not produce significant loss of RI suggesting the activity was relatively heat stable. The RI activity of *P*. *davidi* IAP is also relatively heat stable [6] and so is that of several of the plant AFPs [22]. The level of RI was moderate, with an increase in crystal size during annealing and a reduction in RI after 1:3 dilution, almost to that of a buffer control. Smith *et al* [16] compared the RI activity of six nematode species including an entomopathogenic nematode, *S*. *carpocapsae*. The annealing period and the concentration used (20 min, 18 mg/ml) were different than that in the present study (30 min, 25.3 mg/ml), so the results cannot be directly compared. However, despite the lower concentration and shorter annealing time, the crystal size was smaller in two of the six species (*S*. *carpocapsae* and *P*. *davidi*) than that of the *S*. *feltiae* extract shown here. This places *S*. *feltiae* in a moderate RI category. However, a moderate level of RI may be of importance for this moderately freeze tolerant species which survives intracellular freezing to -3°C [23]. Ramløv *et al* [19] suggest that for a freeze tolerant animal, RI may be important to survive the freezing stress. It plays a role in inhibiting the growth of ice crystals and/or in controlling the size, shape and location of ice crystals after their formation [6, 24]. The moderate RI activity of *S*. *feltiae* may play a role in the size and shape of ice crystals, as indicated by their appearance in freeze-substituted specimens [23]. This protects the nematode from damage and enables it to survive intracellular freezing to -3°C but not to the same extent as *P*. *davidi*, which has strong RI activity and survives to much lower temperatures [14]. However, there is not a direct correspondence between RI activity and nematode survival [14], suggesting that other factors are also important.

The optical transmittance of nematode extract did not change during annealing at -8°C, whereas that of buffer controls and diluted extract increased significantly. There was good correspondence between crystal size in the splat freezing assay and optical transmittance in the recrystallometer in similar samples. This indicates that both assays are detecting recrystallization, although the physical processes involved in the two assays are likely to be different. Recrystallization is both temperature and time sensitive, so both assays are influenced by these factors [18]. Wharton *et al* [20] measured the RI of various samples and reported similar results after comparing the two techniques but they used short annealing times (up to 30 min). The use of much longer annealing times (24 hr) gives more consistent results with the optical recrystallometer and annealing within a refrigerated circulator allows large numbers of samples to be measured. The technique may thus be useful for screening large number of samples, such as column fractions, before confirming RI activity using the splat freezing assay.

No thermal hysteresis was detected in the nematode extract. An hexagonal crystal shape, however, suggests the binding of a substance, most probably a protein, to the ice. In most cases this is due to an antifreeze protein [11, 25]. The lack of thermal hysteresis activity in our nematode sample indicates the absence of an antifreeze protein or its presence in low concentrations [26]. Antifreeze proteins are usually present in freeze avoiding organisms [7], and the absence of thermal hysteresis in a freeze tolerant organism was expected. Duman et al [27] suggest that freezing tolerance may be associated with low, rather than high, levels of thermal hysteresis. However, some freeze tolerant insects have been reported to possess antifreeze proteins [28] and some freeze avoiding animals may have little RI activity [29].

*Steinernema feltiae* had weak ice nucleating activity, allowing the extracts of the nematode to freeze at −14.6°C. However, this activity is unlikely to be involved in the survival of the nematode, as this species can survive freezing down to −3°C at the most. Thus, nucleation of the whole nematode seems to be exogenous as nematodes are essentially aquatic and rely on inoculative freezing [9]. Once the surrounding water is frozen, nematodes freeze by ice inoculation from the surrounding water via natural body openings, such as the secretory-excretory pore [24]. Most freeze tolerant organisms produce ice nucleating agents to ensure freezing at high sub-zero temperature, thus protecting the organism from freezing stress [12]. An extract of the alpine cockroach *Celatoblatta quinquemaculata*, for example, freezes at −5°C having strong nucleators [21]. Conversely, *P*. *davidi*, has no ice nucleating activity, rather produces ice nucleation inhibitors [30]. The weak ice nucleating activity of *S*. *feltiae* is thus probably an incidental property of some of its component molecules or of its intestinal contents.

In conclusion, *S*. *feltiae* has no thermal hysteresis or strong ice nucleating activity. The recrystallization inhibition activity found may assist the freezing tolerance of the nematode but there could be other factors involved, such as production of low molecular weight cryoprotectants [5], that may assist in their freezing survival.

## References

[1] Ramløv H. Aspects of natural cold tolerance in ectothermic animals. Human Reprod. 2000;15(Suppl 5):26–46. 10.1093/humrep/15.suppl_5.26 11263535

[2] Wharton DA. Parasites and low temperatures. Parasitology. 1999;119(SupplementS1):S7–S17. 10.1017/S0031182000084614 11254149

[3] Wharton DA, Goodall G, Marshall CJ. Freezing rate affects the survival of a short-term freezing stress in *Panagrolaimus davidi*, an Antarctic nematode that survives intracellular freezing. CryoLetters. 2002;23(1):5–10. 11912502

[4] Wharton DA, Judge KF, Worland MR. Cold acclimation and cryoprotectants in a freeze-tolerant Antarctic nematode, *Panagrolaimus davidi*. J Comp Physiol B. 2000;170(4):321–7. 10.1007/s003600000106 10935523

[5] Ali F, Wharton DA. Infective juveniles of the entomopathogenic nematode, *Steinernema feltiae* produce cryoprotectants in response to freezing and cold acclimation. PLoS ONE. 2015;10(10):e0141810 10.1371/journal.pone.0141810 26509788 PMC4625012

[6] Wharton DA, Barrett J, Goodall G, Marshall CJ, Ramløv H. Ice-active proteins from the Antarctic nematode *Panagrolaimus davidi*. Cryobiology. 2005;51(2):198–207. 10.1016/j.cryobiol.2005.07.001 . 16102742

[7] Duman JG. Antifreeze and ice nucleator proteins in terrestrial arthropods. Annu Rev Physiol. 2001;63(1):327–57. . 11181959 10.1146/annurev.physiol.63.1.327

[8] Fletcher GL, Hew CL, Davies PL. Antifreeze proteins of teleost fishes. Annu Rev Physiol. 2001;63(1):359–90. . 11181960 10.1146/annurev.physiol.63.1.359

[9] Zachariassen KE, Kristiansen E. Ice nucleation and antinucleation in nature. Cryobiol. 2000;41(4):257–79. 10.1006/cryo.2000.2289 11222024

[10] Wharton DA. Cold tolerance In: Perry RN, Wharton DA, editors. Molecular and physiological basis of nematode survival. Wallingford: CABI Publishing; 2011.

[11] Barrett J. Thermal hysteresis proteins. Int J Biochem Cell Biol. 2001;33(2):105–17. 10.1016/S1357-2725(00)00083-2 11240367

[12] Lee RE, Costanzo JP. Biological ice nucleation and ice distribution in cold-hardy ectothermic animals. Annu Rev Physiol. 1998;60:55–72. 10.1146/annurev.physiol.60.1.55 . 9558454

[13] Knight CA, Duman JG. Inhibition of recrystallization of ice by insect thermal hysteresis proteins: A possible cryoprotective role. Cryobiology. 1986;23(3):256–62. 10.1016/0011-2240(86)90051-9

[14] Knight CA, Wen D, Laursen RA. Nonequilibrium antifreeze peptides and the recrystallization of ice. Cryobiology. 1995;32(1):23–34. 10.1006/cryo.1995.1002 7697996

[15] Ali F, Wharton DA. Cold tolerance abilities of two entomopathogenic nematodes, *Steinernema feltiae* and *Heterorhabditis bacteriophora*. Cryobiology. 2013;66:24–9. 10.1016/j.cryobiol.2012.10.004 23142823

[16] Smith T, Wharton DA, Marshall CJ. Cold tolerance of an Antarctic nematode that survives intracellular freezing: comparisons with other nematode species. J Comp Physiol B. 2008;178(1):93–100. Epub 2007/08/23. 10.1007/s00360-007-0202-3 . 17712562

[17] Greenaway P. Sodium regulation in freshwater mollusc *Limnaea stagnalis* (L) (Gastropoda, Pulmonata). J Exp Biol. 1970;53(1):147–63. . 5478671 10.1242/jeb.53.1.147

[18] Knight CA, Hallett J, DeVries AL. Solute effects on ice recrystallization: An assessment technique. Cryobiology. 1988;25(1):55–60. 10.1016/0011-2240(88)90020-X 3349811

[19] Ramløv H, Wharton DA, Wilson PW. Recrystallization in a freezing tolerant antarctic nematode, *Panagrolaimus davidi*, and an alpine weta, *Hemideina maori* (Orthoptera: Stenopelmatidae). Cryobiology. 1996;33(6):607–13. 10.1006/cryo.1996.0064 . 8975688

[20] Wharton DA, Wilson PW, Mutch JS, Marshall CJ, Lim M. Recrystallization inhibition assessed by splat cooling and optical recrystallometry. CryoLetters. 2007;28(1):61–8. . 17369963

[21] Wharton DA, Mutch JS, Wilson PW, Marshall CJ, Lim M. A simple ice nucleation spectrometer. CryoLetters. 2004;25(5):335–40. Epub 2004/12/25. . 15618985

[22] Sidebottom C, Buckley S, Pudney P, Twigg S, Jarman C, Holt C, et al Heat-stable antifreeze protein from grass. Nature. 2000;406(6793):256 10917518 10.1038/35018639

[23] Ali F, Wharton DA. Intracellular freezing in the infective juveniles of *Steinernema feltiae*: an entomopathogenic nematode. PLoS One. 2014;9(4):e94179 10.1371/journal.pone.0094179 . 24769523 PMC4000207

[24] Wharton DA, Ferns DJ. Survival of intracellular freezing by the Antarctic nematode *Panagrolaimus davidi*. J Exp Biol. 1995;198(6):1381–7. . 9319273 10.1242/jeb.198.6.1381

[25] Jia Z, Davies PL. Antifreeze proteins: an unusual receptor-ligand interaction. Trends Biochem Sci. 2002;27(2):101–6. 10.1016/S0968-0004(01)02028-X 11852248

[26] Davies PL. Ice-binding proteins: a remarkable diversity of structures for stopping and starting ice growth. Trends Biochem Sci. 2014;39(11):548–55. 10.1016/j.tibs.2014.09.005 25440715

[27] Duman JG, Bennett V, Sformo T, Hochstrasser R, Barnes BM. Antifreeze proteins in Alaskan insects and spiders. J Ins Physiol. 2004;50(4):259–66. . 10.1016/j.jinsphys.2003.12.003 15081818

[28] Wharton DA, Pow B, Kristensen M, Ramløv H, Marshall CJ. Ice-active proteins and cryoprotectants from the New Zealand alpine cockroach, *Celatoblatta quinquemaculata*. J Insect Physiol. 2009;55(1):27–31. 10.1016/j.jinsphys.2008.09.007 . 18955061

[29] Block W, Zettel J. Activity and dormancy in relation to body water and cold tolerance in a winter active springtail (Collembola). Eur J Entomol. 2003;100:305–12. 10.14411/eje.2003.049

[30] Wharton DA, Worland MR. Ice nucleation activity in the freezing-tolerant Antarctic nematode *Panagrolaimus davidi*. Cryobiology. 1998;36(4):279–86. .

